# Society Meetings

**Published:** 1905-11

**Authors:** 


### SOCIETY MEETINGS.

THE Lake Erie Medical Society held its last quarterly meeting at the Gowanda State Hospital, October 27, 1905, under the presidency of Dr. Southworth, of Forestville. Dr. B. E. Smith, of Angola, is vice-president and Dr. E. W. James, of Hamburg, is the secretary and treasurer. The program included a paper by Ellis Storms, of Cherry Creek, entitled, Eclampsia; one by Albert D. Lake, of Gowanda, entitled, Something about Tuberculosis; and one by Arthur W. Hurd, of Buffalo, entitled, Korsakoff's Psychosis.

---

THE New York and New England Association of Railway

Surgeons held its fifteenth annual meeting at the Academy of Medicine, New York, November 17, and 18, 1905, under the presidency of G. P. Conn, of Concord, N. H. George Chaffer, of Brooklyn, is the secretary.

---

The Medical Society of the State of New York will hold its one hundredth annual meeting at Albany, January 30, February 1 and 2, 1906. The president, Dr. Joseph D. Bryant, has appointed the following-named business committee: Leo H. Newman, chairman, Albany; A. T. Bristow, Brooklyn; and Herbert U. Williams, Buffalo, either of whom may be addressed concerning the program.

---

The Fifteenth International Medical Congress will be held in Lisbon, from April 19 to 26, 1906, and the committee in charge has written asking for the contribution of papers on the following medico-legal subjects, and saying that as yet no titles of communications touching on any of these subjects have been received from this country:

The signs of virginity and of defloration in medico-legal relations.

Hand marks and finger prints; their medico-legal importance.

The medico-legal importance of the caruncul

The mechanism of death by hanging.

The value of bacteriological examination of vulvo-vaginal discharges in the determination of venereal contagion.

The signs of death by drowning.

Ecchymoses in legal medicine.

Spontaneous and criminal abortion from a medico-legal point of view.

Medico-legal investigation of bloodstains.

The relations between the seat of cerebral contusions and the point of application of the agent which produced them.

Epilepsy in legal medicine.

The induction of abortion, when is it permissible?

The value of legal medicine in the study of criminal law.

The best legislation for the protection of the "medical secret," (the obligation impressed upon physicians to treat as inviolable all information concerning patients obtained while in the discharge of their professional duties).

The effects of the civil and penal law towards the newborn living infant.

Distinction between the natural openings in the hymen and tears of this membrane.

Criminal vulvar copulation.

Organisation of medico-legal services.

If any of the readers of this comunication intend to take part in the discussions of this section of the congress, or to prepare papers for it on any of the subjects mentioned, or on any other subject in medicine or surgery, he should inform the undersigned secretary of the American committee.— Ramon Guiteras, Secy. Am. Nat'l. Com., 75 W. 55th St., New York.

In regard to transportation to the congress Dr. Charles Wood Fassett, St. Joseph, Mo., announces that final arangements have been perfected for the tour of the American party, which will sail on Saturday, April 17, on the North German Lioyd steamer "Koenig Albert" for Gibraltar, visiting Algerciras, Seville, Cordova, etc., spend a week in Lisbon during the congress and return to New York on Wednesday, May 9. This trip may be made comfortably in a first-class steamer both ways, all expenses paid, including board and lodging while in Lisbon, and entertainment at other points, for $300.00.

A number of side trips are being added and the ticket will be good returning through Europe if desired at a slightly increased cost.

Following is a list of those who have booked:

Lewis S. McMurtry, Louisville.

Nicholas Senn, Chicago.

J. D. Griffith, Kansas City.

W. F. Southard, San Francisco.

Frank P. Norbury. Jacksonville, Ill.

W. T. Corlett, Cleveland.

C. H. Hughes, St. Louis.

R. T. Morris, New York.

A. Vander Veer, Albany.

Jos. M. Mathews, Louisville.

J. B. Murphy, Chicago.

Fenton B. Turck, Chicago.

Jas. E. Moore, Minneapolis.

Ramon Guiteras, New York.

Dr. John H. Musser (Philadelphia) is chairman of the American National Committee, and Dr. Ramon Guiteras (75 West 55th street, New York City) is the secretary, to whom all applications for membership and communications in regard to the presentation of papers should be addressed.

All those who contemplate the trip are requested to make reservation with Dr. Fassett at once in order to secure a desirable berth on the steamer and good hotel accommodations. Program of the itinerary upon request.

---

The Western Surgical and Gynecological Association will hold its next annual meeting at Kansas City, December 28 and 29, 1905, under the presidency of Dr. H. D. Niles, of Salt Lake City.

---

The Medical Association of Central New York held a very large and successful meeting in Buffalo, October ,24 1905, under the presidency of Dr. Charles G. Stockton of this city, who chose for the subject of his address: The Nature and Methods of Medical Societies. Officers were elected as follows: president, D. M. Totman, Syracuse; vice-presidents, William B. Jones, Rochester, and L. L. Tozier, Batavia; secretary, C. A. Greenleaf; treasurer, W. M. Brown, the latter two of Rochester. The next meeting will be held at Syracuse.

---

The American Academy of Ophthamology and Otolaryngology which recently held its annual meeting in Buffalo, elected officers for tht ensuing year as follows: president, Casey A. Wood, Chicago; vice-presidents, J. A. Stucky, Lexington, Ky., Alvin A. Hubbell, Buffalo, and Emil Mayer, New York; secretary, George F. Suker, Chicago; treasurer, Otto J. Stein, Chicago.

---

The Homeopathic Medical Society of Western New York held its regular quarterly meeting at Rochester, October 13, 1905. The question of teaching sexual hygiene in the public schools was discussed by Dr. W. S. Rambo, and Rev. Paul M. Strayer, of Rochester; Dr. Dewitt G. Wilcox, of Buffalo, and others.

---

The Erie County Medical Association held its regular quarterly meeting at Buffalo, October 9, 1905. Program: "Suicide", James W. Putnam; discussion opened by A. W. Hurd; dacryocystitis neonatorum, E. E. Blaauw; discussion opened by L. M. Francis. The president is A. G. Bennett and the secretary is David E. Wheeler.

---

The Southern Surgical and Gynecological Association will hold its next annual meeting at the Hotel Seelbach, Louisville, Tuesday, Wednesday and Thursday, December 12, 13, and 14, 1905 under the presidency of Lewis C. Bosher, of Richmond. The secretary is William D. Haggard, of Nashville, and William O.

Roberts, of Louisville, is chairman of the committee of arrangements.

---

THE Buffalo Academy of Medicine held meetings during the month of October as follows:

A stated meeting of the Academy, Tuesday, October 3, 1905, at 8.30 P. M.

A meeting of the council was held at 8.15.

*Section on Surgery.*—Program: Toxemias, surgical and septic fevers, Eugene A. Smith. Discussion opened by Drs. Stockton and Mann.

*Section on Medicine.*—Tuesday, October 10, 1905, at 8.30 P. M. Program: (a) What symptoms really belong to eye strain and what are imaginary? Lucien Howe; (b) Modern methods in typhoid fever, D. W. Harrington.

*Section of Pathology.*—Tuesday, October 17, 1905, at 8.30 P. M. Program: (a) Intestinal tuberculosis in infants, Irving M. Snow. (b) Cutaneous tuberculosis, Charles A. Bentz. Specimens from cases of splenic leukemia, N. L. Burnham.

*Section of Obstetrics and Gynecology.*—Tuesday, October 24, 1905, at 8.30 P. M. Program: The present status of vaginal coesarean section, Martin Stamm, Fremont, O.

